# Serum leucine‐rich alpha‐2 glycoprotein in monitoring disease activity and intestinal mucosal healing for biotherapy‐naïve cases with ulcerative colitis

**DOI:** 10.1002/jgh3.12953

**Published:** 2023-08-03

**Authors:** Masashi Kono, Yoriaki Komeda, George Tribonias, Saki Yoshida, Kenji Nomura, Kohei Handa, Tomoyuki Nagai, Satoru Hagiwara, Shunsuke Omoto, Mamoru Takenaka, Naoshi Nishida, Naoko Tsuji, Hiroshi Kashida, Masatoshi Kudo

**Affiliations:** ^1^ Department of Gastroenterology and Hepatology Kindai University Faculty of Medicine Osaka Japan; ^2^ General Hospital of Nikaia‐Piraeus “Agios Panteleimon” Athens Greece

**Keywords:** biotherapy‐naïve, leucine‐rich alpha‐2 glycoprotein, ulcerative colitis

## Abstract

**Background and Aim:**

Serum leucine‐rich alpha‐2 glycoprotein level has been reported to be a useful biomarker in assessing mucosal healing in patients undergoing biotherapy, where mucosal lesions caused by ulcerative colitis are difficult to assess endoscopically. However, no such reports have been reported in biotherapy‐naïve cases.

**Methods:**

Sixty‐eight patients with ulcerative colitis (UC) who were biotherapy‐naïve at Kindai University Hospital between October 2021 and October 2022 were enrolled. We prospectively examined the correlation between leucine‐rich alpha‐2 glycoprotein (LRG), C‐reactive protein (CRP), erythrocyte sedimentation rate (ESR), and Geboes scores with clinical endoscopic activity using the Mayo endoscopic subscore (MES).

**Results:**

Mucosal healing was achieved in 39 (57%) patients. Univariate analysis revealed that the factors associated with mucosal healing were LRG (*P* = 0.0024), CRP (*P* = 0.1078), ESR (*P* = 0.0372), and Geboes scores (*P* = 0.0075). Logistic regression analysis identified LRG and Geboes scores as independent factors associated with mucosal healing assessed using MES (*P* = 0.0431 for LRG and *P* = 0.0166 for Geboes scores).

**Conclusion:**

LRG was found to be the easiest marker to monitor disease activity and mucosal inflammation in UC patients with biotherapy‐naïve cases, with a performance equivalent to that of Geboes scores.

## Graphical abstract

Leucine‐rich alpha‐2 glycoprotein (LRG) was the best marker to monitor disease activity and mucosal inflammation in patients with UC who had not undergone biotherapy. LRG performance is equivalent to that of Geboes scores.

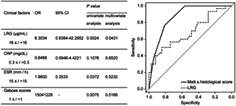

## Introduction

Ulcerative colitis (UC) is one of the most common types of inflammatory bowel disease (IBD).^1^ It is estimated that there are more than 220 000 people with UC in Japan, and these numbers show no signs of decreasing.^2^

The goal of IBD treatment has shifted from clinical remission to endoscopic remission, where endoscopy is often used to assess disease activity. However, frequent endoscopic examinations are not realistic considering the burden on the body and the complexity of the procedure. Therefore, although there are several noninvasive and readily available biomarkers for IBD, their sensitivity and specificity are not sufficient for use as a single marker. Thus, there is an urgent need to identify markers that monitor disease activity.

As for existing markers for monitoring disease activity, C‐reactive protein (CRP) is widely used as a simple biomarker that is detected in serum when there is inflammation in the intestinal tract.^3^, ^4^ Erythrocyte sedimentation rate (ESR) is also used as an indicator of disease activity in IBD because of intestinal inflammation and associated anemia and nutritional disorders.^5^ Fecal calprotectin is required for UC monitoring in STRIDE‐II.^6^ Serada et al. discovered leucine‐rich alpha‐2 glycoprotein (LRG) as a new biomarker for rheumatoid arthritis and IBD in a proteomics approach.^7^ Recently, LRG measurement has been reported to be useful in situations where mucosal lesions in UC are difficult to assess.^8^ In fact, LRG has the same acute‐phase protein properties as CRP, is produced in the liver, and its expression is increased by inflammatory cytokine stimulation. LRG is also induced by cytokines such as interleukin (IL)‐1β, tumor necrosis factor‐alpha (TNF‐α), and IL‐22 in addition to IL‐6, and, unlike CRP, its expression is increased at inflammatory sites.^7^

Existing serum markers, such as CRP and ESR, are often insufficient in cases where only minor clinical symptoms are observed but inflammation in the intestinal tract is confirmed by endoscopy, which is the gold standard. There are some difficulties in evaluating UC disease activity because serum markers such as CRP and ESR are within the reference range. Recently, it was reported that LRG is closely associated with endoscopic activity in UC with biotherapy,^8^, ^9^, ^10^, ^11^ but there have been no such reports in biotherapy‐naïve cases. In this study, we investigated LRG as a biomarker for the presence of UC in biotherapy‐naïve patients.

## Materials and methods

### Study design

This was a prospective observational cohort study performed at Kindai University Hospital in Osaka, Japan. Clinical data were collected prospectively from October 2021 to October 2022.

This study was conducted in accordance with the ethical guidelines of the Declaration of Helsinki revised in 2002 and was approved by the Institutional Review Board (no. 2021–271) of Kindai University Hospital.

### Patients

Sixty‐eight patients with UC who had not previously received biotherapy and who visited Kindai University Hospital between October 2021 and October 2022 were included in the study. The patients in this study were not taking any new drugs, including JAK inhibitors, biologics, or investigational drugs. Four patients were taking concomitant prednisone, 21 patients were taking concomitant thiopurine, and 2 patients were taking both. Two patients were taking prednisone and thiopurine together. Inclusion criteria were patients with UC over the age of 18 who underwent blood tests (LRG, CRP, and ESR) and endoscopy including biopsy at Kindai University Hospital. Exclusion criteria were bowel resection, inappropriate interval between LRG measurement, and endoscopy within 3 months.

### Data collection

Blood sampling (LRG, CRP, and ESR) and endoscopy were performed in separate months, but only results within 3 months of the date of LRG measurement and endoscopy were extracted. Clinical and endoscopic disease activity scores were obtained by collecting serum samples from patients and reviewing their clinical records.

Baseline values for blood tests were as follows: serum CRP (median, 0.40 mg/dL; range, 0.00–15.2 mg/dL), serum LRG (median, 14.7 μg/mL; range, 6.5–65.8 μg/mL), platelet count (median, 26.5 × 10^4^/μL, range, 12.9–51.7 × 10^4^/μL), and ESR (median, 16.8 mm/h, range, 1–116 mm/h).

Baseline values for blood tests in patients with UC who had not previously received a biologic are as follows: serum CRP (median, 0.24 mg/dL; range, 0.00–4.0 mg/dL), serum LRG (median, 13.9 μg/mL; range, 6.5–37.9 μg/mL), platelet count (median, 25.9 × 10^4^/μL, range, 12.9–51.7 × 10^4^/μL), and ESR (median, 15.2 mm/h, range, 1–72 mm/h).

The normal ranges for the various blood tests were adopted from those at our hospital and were divided into two groups: one within the normal range, and the other outside the normal range, for significant difference tests (Table 1). Using endoscopy, the Mayo endoscopic subscore (MES)^12^ was collected for each patient, whose median was 1.24 and range 0–3 (Table 1). The Mayo scores for the 68 cases were as follows: MES0 in 17 patients, MES1 in 21, MES2 in 25, and MES3 in 5. The mean Mayo score for the 68 cases was 5.05.

**Table 1:** Association between biomarkers and mucosal healing in cases with ulcerative colitis with biotherapy‐naïve cases

Clinical factors	OR	95% CI	*P‐*value	
			Univariate analysis	Multivariate analysis
LRG (μg/mL) 16 ≤/> 16	6.3034	0.9394–42.2952	0.0024	0.0431
CRP (mg/dL) 0.3 ≤/> 0.3	0.6468	0.0946–4.4221	0.1078	0.6520
ESR (mm/h) 15 ≤/> 15	1.6600	0.3533	0.0372	0.5232
Geboes scores 1 ≤/> 1	15 041 228	‐	0.0075	0.0166

CI, confidence interval; OR, odds ratio.

### Pathologic evaluation

In the histologic studies, a certificated pathologist evaluated the Geboes scores.^13^ Histologic disease activity of UC was classified into six grades (grades 0–5). The biopsy specimen was taken from the site with maximum endoscopic activity, and histologic remission was defined as a Geboes score ≤1. The histologic remission group was set to MES0 and 1, while the non‐histological remission group was set to MES2 and 3.

Biopsy specimens were read by two independent pathologists who were blinded to the patients' condition and endoscopic results. In case of disagreement between the two pathologists, a third was consulted.

### Endpoints

The primary endpoint was the correlation between the biomarker (LRG, CRP, and ESR) and endoscopic activity (mucosal healing), and the secondary endpoint was the correlation between Geboes scores and endoscopic activity (mucosal healing).

### Statistical analysis

We wanted to determine whether there was a significant correlation between the presence of endoscopic mucosal healing using the MES and blood serum levels including LRG. A statistical analysis was performed to identify the predictors associated with mucosal healing or mucosal non‐healing for all patients with UC. Endoscopic mucosal healing was defined as MES0 and 1 and mucosal non‐healing as MES2 and 3.

In the univariate analysis, CRP, LRG, ESR, and Geboes scores were treated as categorical variables. Pearson's chi‐square test was applied to compare categorical variables. Baseline factors that differed significantly in the univariate analysis were analyzed using multiple logistic regression analysis. All *P*‐values were two‐tailed, and *P* < 0.05 was considered to show statistical significance. Statistical analysis was performed by the Easy R software (available at https://www.jichi.ac.jp/saitama-sct/SaitamaHP.files/windowsEN.html).

## Results

### Characteristics of the enrolled patients

The patient characteristics are presented in Table 2. Among the patients with UC attending our hospital, we selected those who had undergone colonoscopy and had their LRG levels recorded. We excluded patients who were <18 years of age or who were transferred to other hospitals. Finally, 68 patients with UC for whom LRG measurement and endoscopy were appropriate were considered. There were 38 men and 30 women (median age 49 years, range 19–86 years). Thirty‐five patients had total colitis and 33 had left or rectal colitis. The mean disease duration was 10.7 (1–38) years. Treatment consisted of steroids in 12 cases and thiopurines in 18 cases. Clinical characteristics were associated with mucosal healing according to MES and LRG, CRP, ESR, and Geboes scores in biotherapy‐naïve patients. Sixty‐eight patients who did not have a history of biologic therapy were selected for analysis and divided into two groups: mucosal healing and non‐mucosal healing groups (Table S1).

**Table 2:** Baseline clinical characteristics of patients with ulcerative colitis with biotherapy‐naïve cases

Clinical factors	Baseline characteristics
Sex (male/female)	38/30
Median age (year) (range)	49 (19–86)
Median CRP index (mg/dL) (range)	0.24 (0.00–4.0)
Median LRG index (μg/mL) (range)	13.9 (6.5–37.9)
Median PLT level (×10^4^/μL) (range)	25.9 (12.9–51.7)
Median ESR level (mm/h) (range)	15.2 (1–72)
Median Mayo endoscopic subscore (range)	1.24 (0–3)
Type of UC (pancolitis/non‐pancolitis)	35/33
5‐ASA (with/without)	64/4
Combined with PSL (with/without)	12/56
Combined with thiopurine (with/without)	18/50
Median disease period (year) (Range)	10.7 (1–38)

Number of the patients or median value (range) is shown.

5‐ASA, 5‐aminosalicylic acid; CRP, c‐reactive protein; ESR, erythrocyte sedimentation rate; LRG, leucine‐rich alpha 2 glycoprotein; PLT, platelet; PSL, prednisolone; UC, ulcerative colitis.

### Association between biomarkers and mucosal healing in cases with ulcerative colitis with biotherapy‐naïve cases

Thirty‐nine (57%) patients achieved mucosal healing. Pearson's chi‐square test was performed on the 68 patients, and univariate analysis revealed that the factors associated with mucosal healing were LRG (*P* = 0.0024), CRP (*P* = 0.1078), ESR (*P* = 0.0372), and Geboes scores (*P* = 0.0075) (Table 1). Logistic regression analysis identified LRG and Geboes scores as independent factors associated with mucosal healing (*P* = 0.0431 for LRG and *P* = 0.0166 for Geboes scores).

**Figure 1:**
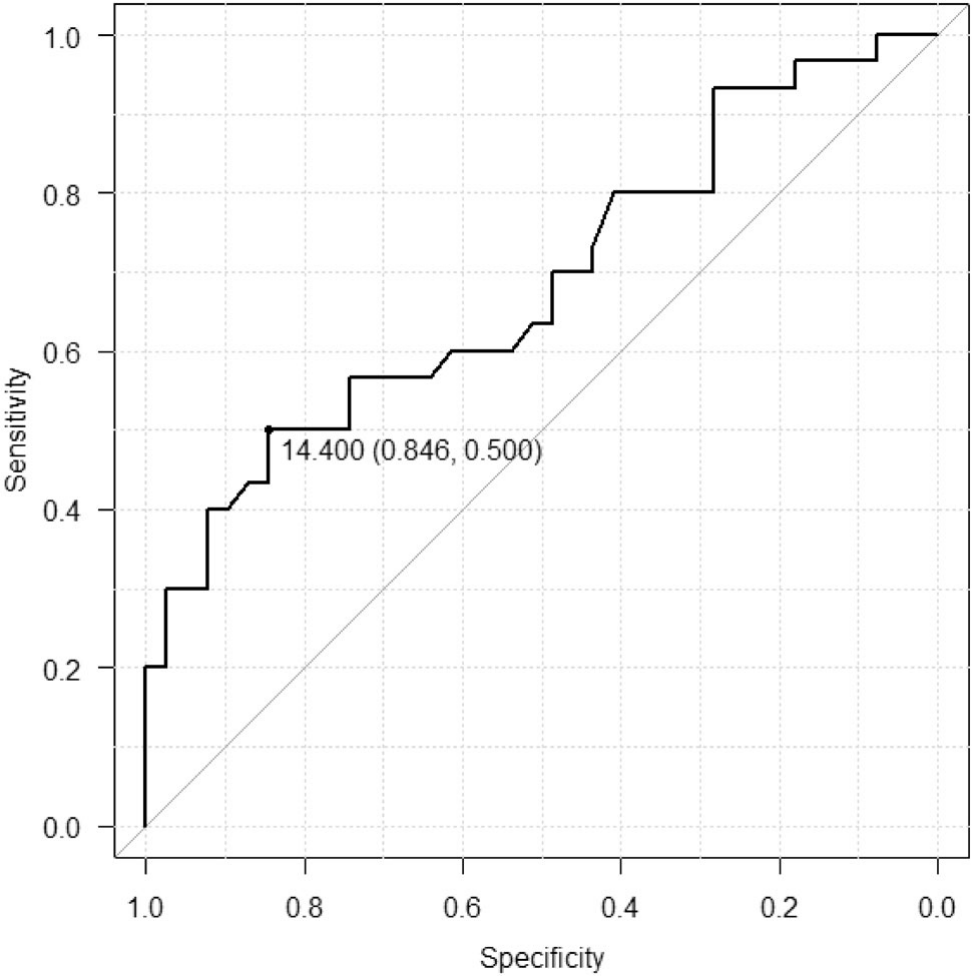
Receiver operating characteristic curves for leucine‐rich alpha‐2 glycoprotein in patients with ulcerative colitis with biotherapy‐naïve cases.

**Figure 2:**
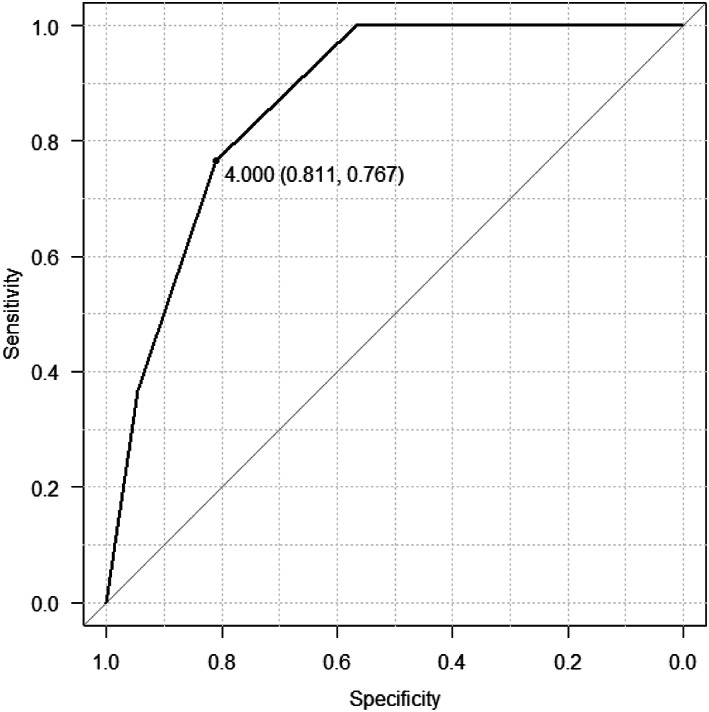
Receiver operating characteristic curves for Geboes scores in patients with ulcerative colitis with biotherapy‐naïve cases.

Thus, LRG and Geboes scores were the factors significantly associated with mucosal healing in patients with UC in the group without prior biologic therapy, and LRG was the best biomarker among the items from the blood test. LRG and Geboes scores were significantly associated with mucosal healing in the group with no prior biologic therapy. Therefore, receiver operating characteristic (ROC) curves were drawn to determine the sensitivity and specificity of LRG (Fig. 1) and Geboes scores (Fig. 2).

**Figure 3:**
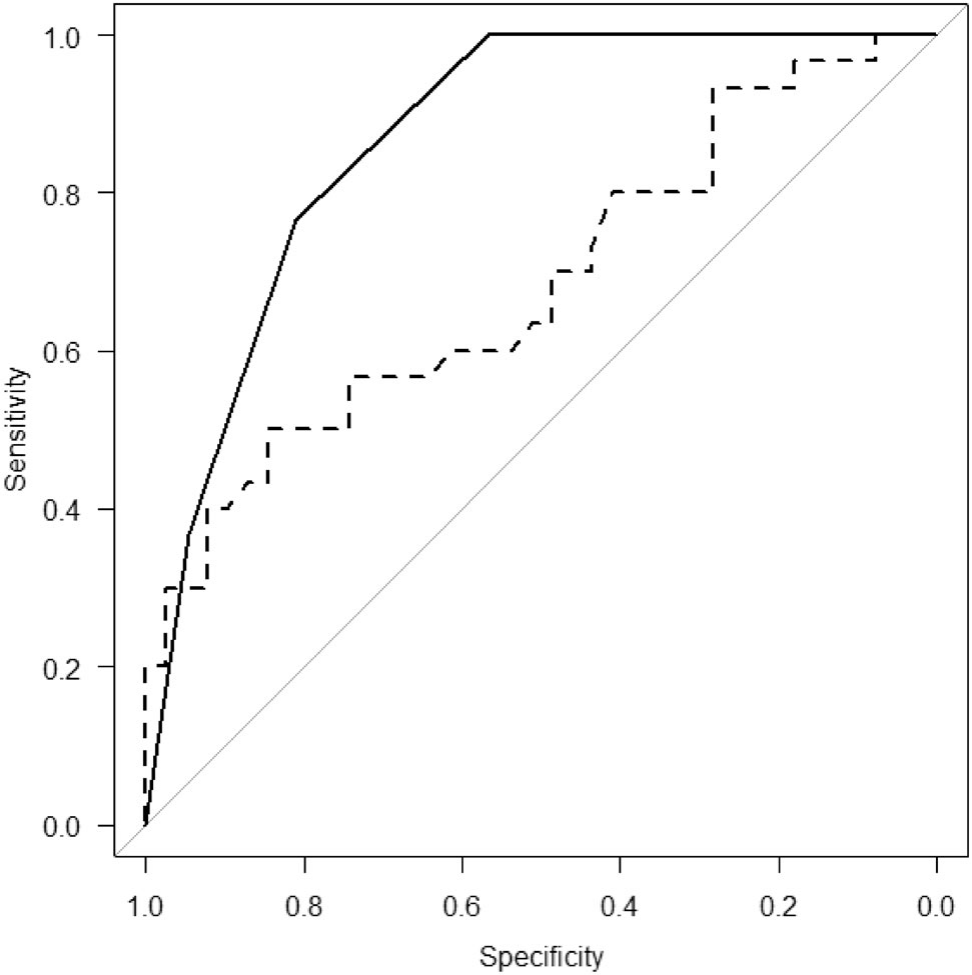
Receiver operating characteristic curves for leucine‐rich alpha‐2 glycoprotein (LRG) and Geboes scores in patients with ulcerative colitis with biotherapy‐naïve cases. 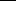, Matt.s.histological.score; 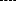, LRG.

ROC curves were related to LRG and Geboes scores in mucosal healing in biotherapy‐naïve cases. As a result, it was estimated that LRG less than 14.4 μg/mL could be judged as mucosal healing with an area under the curve (AUC) of 0.67, sensitivity of 84.6%, specificity of 50.0%, positive predictive value (PPV) of 79.0%, and negative predictive value (NPV) of 59.0%. Geboes scores <4 could be judged as mucosal healing with an AUC of 0.869, sensitivity of 81.8%, specificity of 76.7%, PPV of 82.7%, and NPV of 74.8% (Figs. 1, 2, 3).

Our results confirmed that LRG with a cut‐off value of 14.4 μg/mL is a novel biomarker for predicting the presence of active UC with biotherapy‐naïve cases.

## Discussion

In STRIDE‐II, normalization of the CRP is short‐term goal since CRP has been a validated biological marker for IBD^6^. In addition to CRP, ESR is often used to assess the severity of IBD.^14^ CRP and ESR are substances produced when inflammation occurs in the body and can be used as an indication of UC activity. Transcriptional activity of CRP is mainly induced by IL‐6 stimulation and enhanced by IL‐1. Existing serum markers, such as CRP and ESR, are often insufficient in identifying cases where inflammation of the intestinal tract is shown endoscopically but only minor clinical symptoms are seen. Thus, endoscopy is an essential examination for patients with UC and is a very useful method for assessing disease activity. However, there are some disadvantages, such as the use of laxatives during endoscopy and the physical burden of endoscopy itself. Therefore, there is a possibility that the number of endoscopic examinations can be reduced even slightly if the mucosa is in a healing state, and further study is required to identify which cases fall under this category.

In previous reports on UC with biologic therapy, LRG levels were seen to correlate with the levels of disease activity evaluated by endoscopy, making it possible to easily evaluate the disease activity with a less invasive test.^7^, ^8^ However, the role of LRG is unclear in patients with UC who have not received biotherapy. In addition, the relationship between endoscopic mucosal healing and histologic healing is unclear.

We prospectively examined the correlation between serum LRG, CRP, and ESR levels, as well as Geboes scores, with clinical endoscopic activity according to MES. LRG and Geboes scores were the factors significantly associated with mucosal healing according to MES in patients with UC who have not received biotherapy. LRG was the best biomarker among the blood items in UC with biotherapy‐naïve cases. Recent reports have indicated that complete mucosal healing with MES0 was superior to MES1 in efficacy in predicting recurrence.^15^, ^16^

LRG has the potential to become a biomarker comparable to MES in patients with UC with biotherapy‐naïve cases. Our results confirmed that LRG with a cut‐off value of 10.8 μg/mL is a novel biomarker for predicting the presence of active UC in biotherapy‐naïve cases. Using this cut‐off value, LRG serum levels allow us to identify UC cases with a sensitivity of 84.6% and specificity of 50% (Fig. 1).

This study has some limitations. First, this was a study with a relatively small sample size. However, this small sample size was sufficient for multivariate analysis. Second, our single‐center analysis may have led to selection bias for inclusion of patients. Further studies using a larger sample size in multiple centers are needed to overcome this limitation. Third, fecal calprotectin was not routinely examined. Fecal samples are associated with limited and cumbersome sampling and quantitative capability. However, our results have shown that LRG serum levels could conveniently be obtained with only a blood sample.

## Conclusion

In conclusion, LRG was found to be the best marker to monitor disease activity and mucosal inflammation in patients with UC who had not undergone biotherapy. Performance of LRG is equivalent to that of Geboes scores. However, further studies on the serum marker efficiency of LRG are required before its use in clinical practice of IBD.

## Ethics approval

This study was approved by the Institutional Review Board (no. 2021–271) of Kindai University Hospital. Consent was obtained from all patients in this study.

## Supporting information


**Table S1.** Baseline clinical characteristics of the pathologic mucosal healing and non‐mucosal healing groups of biotherapy‐naive patients with ulcerative colitis.
