# Supplementary material for: Serum leucine‐rich alpha‐2 glycoprotein in monitoring disease activity and intestinal mucosal healing for biotherapy‐naïve cases with ulcerative colitis
Source: JGH Open. 2023 Aug 3;7(8):579–83. doi: 10.1002/jgh3.12953 (PMC10463021; doi:10.1002/jgh3.12953)
Supplement: Supplementary file 1 — Table S1. Baseline clinical characteristics of the pathologic mucosal healing and non‐mucosal healing groups of biotherapy‐naive patients with ulcerative colitis. [file JGH3-7-579-s001.docx]

Supplementary Table. Baseline clinical characteristics of pathologic mucosal healing and non-mucosal healing groups of biotherapy-naive patients with ulcerative colitis

| Clinical factors | Baseline characteristics of mucosal healing | Baseline characteristics of non-mucosal healing |
| --- | --- | --- |
| Sex (Male/Female) | 4 / 6 | 34 / 24 |
| Median Age (year) (Range) | 55, 44-81 | 48.2, 19-86 |
| Median CRP index (mg/dL) (Range) | 0.05, 0.00-0.127 | 0.27, 0.00-4.03 |
| Median LRG index (μg/mL) (Range) | 12.1, 7.3-15.1 | 14.3, 6.5-37.9 |
| Median PLT level (×10^4^/μL) (Range) | 22.9, 12.9-31.9 | 26.4. 14.9-51.7 |
| Median ESR level (mm/h) (Range) | 10, 5-17 | 16.2, 1-72 |
| Median Mayo endoscopic subscore (Range) | 0.18, 0-1 | 1.44, 0-3 |
| Type of UC (pancolitis/non pancolitis) | 4 / 6 | 31 / 27 |
| 5-ASA (with/without) | 8 / 2 | 56 / 2 |
| Combined with PSL (with/without) | 0 / 10 | 12 / 46 |
| Combined with Thioprine (with/without) | 2 / 8 | 16 / 42 |
| Median disease period (year) (Range) | 13 (3 – 31) | 10.3 (1 - 38) |

^†^ Number of the patients or median value (range) was shown.

CRP = c-reactive protein, LRG = Leucine-rich alpha 2 glycoprotein, PLT = platelet, ESR = erythrocyte sedimentation rate, UC = ulcerative colitis

5-ASA = 5-aminosalicylic acid, PSL = prednisolone
